# Economic Burden of Type 2 Diabetes Mellitus Among Patients Attending Ho Teaching Hospital in the Ho Municipality, Ghana: A Cross‐Sectional Study

**DOI:** 10.1002/hsr2.72417

**Published:** 2026-04-22

**Authors:** Maxwell Ayindenaba Dalaba, Phidelia Theresa Doegah, Mustapha Immurana, Robert Kaba Alhassan, Paul Welaga, Michel Adurayi Amenah, Isaiah Awintuen Agorinya, Martin Amogre Ayanore, Paul Amuna, William Kofi Bosu

**Affiliations:** ^1^ Institute of Health Research University of Health and Allied Sciences Ho Volta Region Ghana; ^2^ School of Public Health C.K. Tedam University of Technology and Applied Sciences Navrongo Upper East Region Ghana; ^3^ Social Science Department Ghana College of Physicians and Surgeons Accra Greater Accra Region Ghana; ^4^ FNB School of Public Health University of Health and Allied Sciences hohoe Volta Region Ghana; ^5^ School of Biomedical Sciences University of Cape Coast Cape Coast Central Region Ghana

**Keywords:** catastrophic health expenditure, cost‐of‐illness, diabetes, economic burden, Ghana

## Abstract

**Background and Aims:**

Type 2 diabetes mellitus (T2DM) imposes a growing economic burden in Ghana, yet evidence on its financial impact is limited. This study assessed the economic burden of T2DM among patients receiving outpatient care at Ho Teaching Hospital.

**Methods:**

A cross‐sectional cost‐of‐illness study was conducted between July and December 2024 among 118 T2DM patients. Data were collected using a structured questionnaire developed from a literature review and expert input and administered by trained student nurses. Monthly costs were estimated, and catastrophic health expenditure (CHE) was assessed using the budget share method at 10%, 25%, and 40% income thresholds, applying the 2024 national monthly minimum wage as a proxy. Logistic regression was used to identify predictors of CHE.

**Results:**

Most respondents (88.98%) were enrolled in the National Health Insurance Scheme (NHIS). The mean monthly cost of care was USD21.25, with direct medical costs (USD18.71) accounting for 88% of total expenditure, largely driven by medications (67.17%). Indirect costs due to productivity losses were minimal (USD0.40), although patients spent considerable time traveling (3.2 h) and at the facility (2.4 h) per visit. CHE prevalence was high: 100% at the 10% threshold, 95.76% at 25%, and 88.14% at 40% based on total costs. Older age (40–59 years), unemployment, and lack of tertiary education significantly increased CHE risk, while NHIS enrollment reduced risk at higher thresholds.

**Conclusion:**

Despite high NHIS coverage, T2DM care at Ho Teaching Hospital places a substantial financial burden on patients, with widespread catastrophic expenditure. Expanding NHIS benefits for tertiary diabetes services and reducing time and transport costs are essential to lessen the economic impact of T2DM in Ghana.

**Abbreviations**
CHE: catastrophic Health Expenditure
CHPS: community‐based Health Planning and Services
COI: cost‐of‐illness
HTH: Ho Teaching Hospital
NHIS: National health insurance scheme
OOPs: out‐of‐pocket payments
T2DM: type 2 diabetes mellitus
UHAS: University of Health and Allied Sciences
WHO: World Health Organization

## 1 Introduction

Diabetes mellitus (DM) is a chronic metabolic disorder characterized by abnormally high blood sugar levels. This condition arises from either insufficient insulin production or the body's ineffective use of the insulin it produces [1, 2]. Type 2 diabetes mellitus (T2DM), the most prevalent form, occurs when the body becomes resistant to insulin or does not produce enough, leading to glucose accumulation in the blood. While Type 1 diabetes is an autoimmune condition, T2DM is often linked to lifestyle factors and genetics [1, 2].

Ghana is experiencing a steady increase in T2DM prevalence, driven by rapid urbanization, changing lifestyles such as poor dietary habits, and physical inactivity [3, 4]. It was reported by the WHO that about 2.4 million Ghanaians live with diabetes, with about 7.5% of adults having T2DM [5]. In the Volta region of Ghana, the prevalence of T2DM has been reported to be 6.9% [6].

Despite Ghana's structured three‐tier healthcare system, the management of T2DM remains suboptimal. Recent evidence indicates that nearly 68% of Ghanaian adults with T2DM have poor glycaemic control, significantly increasing the risk of costly microvascular and macrovascular complications [7].

While the National Health Insurance Scheme (NHIS) was designed to move the country toward universal health coverage, its impact on chronic disease management is severely limited. Frequent medication stockouts, weak supply chain systems, and gaps in coverage for essential diagnostics and specialized care mean that the financial protection intended by the NHIS is often illusory [8, 9].

The economic consequence of these systemic gaps is a heavy reliance on out‐of‐pocket (OOP) spending. Estimates for the annual cost of managing diabetes in Ghana vary widely, ranging from $38.68 to $290.44 per patient [10, 11]. These costs extend beyond direct medical fees to include significant nonmedical expenses, such as transportation, and indirect costs associated with lost productivity. For many households, these cumulative expenses result in catastrophic health expenditure (CHE), which threatens to entrench families in poverty or force the cessation of life‐saving treatment [12].

While the clinical burden of diabetes is well‐documented, there is a dearth of localized evidence regarding the specific economic pressures faced by patients at the tertiary level of care. Understanding the financial trajectory of patients at a major referral center is essential for refining policy and improving economic risk protection. Therefore, this study assessed the economic burden of T2DM among patients at the Ho Teaching Hospital. Specifically, it examined the direct and indirect costs, the time burden of accessing services, and the prevalence of CHE among outpatients receiving routine care.

## 2 Methods

### 2.1 Study Design

We conducted a facility‐based, cross‐sectional, cost‐of‐illness (COI) study from the patient perspective to assess the economic burden of T2DM between July and December 2024. This approach allowed us to capture both direct and indirect costs associated with diabetes care, including out‐of‐pocket payments and productivity losses, as experienced by outpatients attending routine outpatient services.

### 2.2 Study Setting

The study was carried out at Ho Teaching Hospital (HTH) in the Ho Municipality of Ghana's Volta Region. As a major public tertiary facility and the fifth teaching hospital in Ghana, HTH serves as the primary referral center for the Volta Region and its adjoining districts. It has a bed capacity of 320 and a workforce of over 650 healthcare professionals. We selected this facility because of its high patient load and its strategic role in managing chronic conditions such as diabetes under the NHIS. The hospital's Diabetic Unit, which offers weekly outpatient clinics and manages a wide range of diabetes cases, was chosen as the study site. This setting provided a suitable environment for examining the economic burden faced by individuals seeking specialized diabetes care in a tertiary hospital context.

### 2.3 Study Population and Eligibility Criteria

Our target population comprised adult patients diagnosed with T2DM who were receiving routine outpatient care at the Diabetic Unit of HTH during the study period. We focused on individuals attending the facility for follow‐up consultations, laboratory investigations, and medication refills related to diabetes management.

To be eligible for inclusion in the study, participants had to meet the following criteria: (1) be 18 years of age or older, (2) have a confirmed clinical diagnosis of T2DM, and (3) be receiving outpatient care at the Diabetic Unit during the data collection period between July and December 2024. We included patients who were registered in the facility's diabetes outpatient registry and were available and willing to provide informed consent on clinic days.

We excluded patients who had a diagnosis of Type 1 diabetes, gestational diabetes, or other forms of diabetes. We also excluded patients who were acutely ill, admitted as inpatients, or those with cognitive impairments that could hinder their ability to understand and respond to the questionnaire. These exclusion criteria were applied to ensure that our findings reflected the typical outpatient diabetes management experience and the associated economic burden among stable T2DM patients.

### 2.4 Sample Size Determination

We determined the required sample size using Cochran's formula for estimating a single population proportion, which is appropriate for cross‐sectional studies. The formula is expressed as:

$$n=\;\frac{Z^{2}*p*(1-p)}{d^{2}},$$

where n is the sample size, Z is the standard normal deviation corresponding to 95% confidence level (1.95), p is the estimated proportion of the population with the characteristic of interest, and d is the desired margin of error.

For this study, we used a prevalence (*p*) of 0.069 (6.9%), based on the reported prevalence of diabetes in the Volta region [6]. To account for potential non‐response, a 20% non‐response rate was applied, resulting in a final adjusted sample size of approximately 118 respondents. This sample size was deemed adequate to ensure sufficient statistical power to estimate the economic burden of diabetes care with reasonable precision among patients attending outpatient services at HTH.

### 2.5 Sampling Strategy

We employed a systematic sampling technique to select participants for the study. The sampling was conducted at the Diabetic Unit of HTH, which records an estimated 163 outpatient visits per month for diabetes care. Given our target sample size of 118 patients and a planned data collection period of 3 months, we anticipated approximately 489 patient visits during the study period. To achieve a representative sample, we calculated a sampling interval by dividing the estimated total number of patient visits (489) by the sample size (118), resulting in a sampling interval of approximately 4.

Using this interval, starting with the first eligible patient in the consultation queue, every fourth eligible patient attending the diabetes clinic was approached for participation. This procedure was repeated each clinic day until the required sample size was achieved. In instances where a selected patient declined to participate or did not meet the eligibility criteria, the next eligible patient was recruited in their place to maintain the sampling interval. This approach ensured a systematic and unbiased selection of participants across the data collection period.

### 2.6 Data Collection Procedures

Data were collected using a structured questionnaire specifically developed to capture the economic burden of diabetes care. The tool was designed to gather information on socio‐demographic characteristics, direct medical and nonmedical costs, and indirect costs related to time lost during care‐seeking. The questionnaire was reviewed by experts in health economics and piloted with a small group of diabetic patients (*n* = 10) at a nearby health facility to ensure clarity, contextual relevance, and cultural appropriateness. Feedback from the pilot informed minor revisions before full deployment.

The finalized questionnaire was programmed into REDCap, a secure, electronic data capture platform, and administered using tablet devices. Data collection was conducted by five student nurses from the University of Health and Allied Sciences (UHAS), Ho, Ghana. Before data collection commenced, all the data collectors were trained on the study protocol, research ethics, and standardized interviewing techniques, and consistent translation of questions into Ewe and Twi.

Interviews were conducted in participants’ preferred languages: English, Ewe, or Twi to ensure comprehension and accurate responses. To strengthen the internal validity of responses obtained in local languages, questionnaires were administered by trained data collectors fluent in both English and the respective local languages.

Direct medical costs were assessed by asking respondents to estimate their OOPs payments incurred in the month preceding the survey, including costs from multiple visits for diabetes management, including consultation fees, medications, and laboratory costs (including HbA1c, lipid profiles, wound care, and imaging test etc). Direct non‐medical costs, primarily transportation expenses, were based on OOP payments incurred during the current hospital visit, which included OOPs for transportation to and from the healthcare facility.

Indirect costs were estimated using the time spent traveling to the hospital and the time spent waiting to receive care during the current visit. Respondents were also asked to estimate the time they expected to spend at the hospital to determine waiting times for care.

### 2.7 Data Analysis

Data were analyzed with Microsoft Excel and STATA version 14. Descriptive statistics, including frequencies and percentages, were used to summarize socio‐demographic characteristics. Both mean and median costs were reported for each cost category.

### 2.8 Cost Estimation Approach

We estimated the economic burden of T2DM from the patient perspective, focusing on three cost categories in line with standard health economic evaluation methods: direct medical costs, direct nonmedical costs, and indirect costs.

The total treatment cost was calculated by summing direct and indirect costs.

Direct medical costs captured all OOPs made by patients for consultations, laboratory investigations, and medications. Direct non‐medical costs consisted of transportation expenses incurred to and from the healthcare facility.

Indirect costs, representing productivity losses, were estimated using the human capital approach, which calculates the economic value of time lost while seeking care [10, 13, 14]. Specifically, we multiplied the total hours spent traveling and waiting at the facility by Ghana's 2024 national daily minimum wage (GHS18.15) [15], and based on an 8‐h workday.

The equivalent in US dollars was calculated using an average exchange rate of USD1 = GHS 15, in line with Bank of Ghana exchange rates for July to September 2024 [16].

### 2.9 CHE Estimation

CHE refers to health‐related spending that threatens a household's ability to meet its basic subsistence needs. In this study, CHE was estimated using the Budget Share (BS) method, the most widely applied approach in the literature [17, 18]. CHE was assessed by calculating the share of a patient's monthly diabetes‐related costs relative to a standardized income benchmark. CHE was determined using the standard formula:

$$\mathrm{CHE}=\left(\frac{\mathrm{Out}\;\mathrm{of}\;\mathrm{pocket}\;\mathrm{health}\;\mathrm{expenditure}}{\mathrm{Individual}\;\mathrm{income}}\right)\;*\;100\;\geq \mathrm{Threshold}\;.$$

In the absence of reliable income data within the study sample, individual income was proxied using Ghana's 2024 national monthly minimum wage of GHS544.50 (USD36.30). This approach was adopted due to widespread informal employment and the lack of disaggregated income reporting in the sample, making self‐reported income unreliable for calculating CHE [19].

CHE was computed separately for two categories of costs: (1) total costs (sum of direct and indirect diabetes‐related expenses), and (2) direct costs alone. To evaluate the robustness of the estimates and capture varying degrees of financial burden, a sensitivity analysis was performed using multiple CHE thresholds of 10%, 25%, and 40% thresholds to capture varying levels of economic burden [13, 20, 21]. For each threshold and cost category, we estimated the proportion of patients exceeding the threshold and reported 95% CIs.

### 2.10 Sensitivity Analysis of Cost Drivers

A one‐way sensitivity analysis was conducted by increasing direct medical costs and indirect costs by 5% relative to total costs to examine their effects on CHE. This analysis assessed the robustness of the CHE estimates and evaluated how changes in cost components and assumptions about financial catastrophe influenced the estimated economic burden of diabetes care.

### 2.11 Regression Analysis

Logistic regression models were then used to explore socio‐demographic predictors of CHE under each specification. For each model, we estimated the average marginal effects (AMEs) along with 95% confidence intervals (CIs) to provide interpretable estimates of the change in probability of experiencing CHE associated with each predictor.

### 2.12 Ethical Consideration

Ethical approval for this study was obtained from the UHAS Research Ethics Committee (Clearance Number: UHAS‐REC A.11 [22] 23–24). Written informed consent was obtained from each respondent prior to the administration of the questionnaires.

## 3 Results

### 3.1 Socio‐Demographic Characteristics of Respondents

A total of 118 respondents participated in the study. The age distribution showed that the largest proportion of participants were between 40 and 49 years and 60 years or older, with each group representing 29.66% of the sample. Females constituted a slightly higher proportion of the study population (52.54%) compared to males (47.46%). In terms of educational attainment, 38.14% of respondents had attained secondary education, while 32.20% had tertiary education. Those with basic education accounted for 20.34%, and 9.32% had no formal education.

Most respondents were married (60.17%), followed by those who were widowed (18.64%), single (12.71%), or divorced (8.47%). Regarding employment status, the majority were self‐employed (37.29%), followed by civil servants (29.66%). The unemployed accounted for 16.95%, while retired individuals constituted 16.10% of the sample.

Housing arrangements varied among respondents, with 39.83% residing in owned homes and 37.29% in rented accommodations. An additional 21.19% lived with family members, while 1.69% reported other types of living arrangements. In terms of health insurance status, a large majority (88.98%) were enrolled in the NHIS, whereas 11.02% were not covered (Table 1).

**Table 1:** Socio‐demographic characteristics of respondents.

Variable	Frequency (*n* = 118)	Percent (100%)
Age (years)		
18–29	4	3.39
30–39	19	16.10
40–49	35	29.66
50–59	25	21.19
60 years	35	29.66
Sex		
Female	62	52.54
Male	56	47.46
Education		
No formal education	11	9.32
Basic education	24	20.34
Secondary	45	38.14
Tertiary	38	32.2
Marital status		
Divorce	10	8.47
Married	71	60.17
Single	15	12.71
Widowed	22	18.64
Occupation		
Civil Servant	35	29.66
Retired	19	16.10
Self‐employed	44	37.29
Unemployed	20	16.95
Residence		
Family	25	21.19
Other	2	1.69
Owned	47	39.83
Rented	44	37.29
NHIS enrollment		
No	13	11.02
Yes	105	88.98

### 3.2 Cost of Diabetes Management

The total cost of managing Type 2 diabetes among study respondents was composed of both indirect and direct cost components (Table 2).

**Table 2:** Cost of diabetes management (USD).

Variable	Mean	Median	Min	Max	% of total cost
Indirect cost					
Productivity lost	0.40	0.38	0.09	0.81	1.88
Direct cost					
Direct nonmedical					
Transport	2.30	1.71	0.33	11.02	10.08
Direct medical cost					
Consultation	2.75	2.75	2.75	2.75	1.21
Medicine	14.28	13.77	0.28	44.08	67.17
Lab	4.18	5.51	0.28	6.61	19.66
Subtotal‐medical cost	18.71	17.49	3.86	50.14	88.04
Subtotal‐direct costs	20.85	19.34	4.41	53.44	98,12
Grand total‐direct and Indirect costs	21.25	19.79	4.50	53.94	100

**Indirect costs**: Indirect costs, primarily due to productivity losses from time spent seeking care, were estimated at an average of USD0.40 per visit (Median = USD0.38). These costs are relatively low, contributing only 1.88% to the total cost of illness

**Direct costs:** Direct costs, comprising both medical and non‐medical expenses, accounted for an average of USD20.85 per month (Median = USD 19.34), representing 98.12% of the total treatment cost.

***Direct non‐medical costs***: These costs, mostly for transportation to and from the health facility, averaged USD2.30 per visit (Median = USD 1.71), making up 10.08% of the total cost. On average, the time spent traveling to and from the facility by respondents was 192.19 min (3.2 h) (Median = 180 min/3 h). In addition, the time spent at the facility was 141.47 min (Median = 120 min).

**Direct medical costs:** On average, patients incur a direct medical cost of USD18.71 (median = USD17.49) per month, which included USD2.75 (Median = USD2.75) for consultations, USD14.28 (Median = USD13.77) for medicines, and USD4.18 (Median = USD5.51) for Laboratory investigations. Medicines constituted the largest share of expenses, accounting for 67.17% of the total treatment cost.

**Overall cost**: The total cost, including both direct and indirect components, was estimated at an average of USD21.25 per month (Median = USD19.79). When extrapolated annually, the cost amounts to approximately USD255.05 per patient per year (Median = USD 237.47).

### 3.3 CHE

Table 3 reports the incidence of CHE at thresholds of 10%, 25%, and 40% for both total and direct healthcare costs.

**Table 3:** Catastrophic health expenditure (CHE) by threshold and cost type.

Cost category	CHE threshold (%)	CHE status	Frequency (*n* = 118)	Percent (%)
Total cost	40	No	14	11.86
		Yes	104	88.14
	25	No	5	4.24
		Yes	113	95.76
	10	No	0	0
		Yes	118	100
Direct medical cost	40	No	25	21.19
		Yes	93	78.81
	25	No	0	0
		Yes	118	100
	10	No	0	0
		Yes	118	100

CHE was highly prevalent across all thresholds. Based on total costs, 88.1% of patients experienced CHE at the 40% threshold, increasing to 95.8% at 25%, and reaching 100% at the 10% threshold. For direct medical costs alone, 78.8% of patients incurred CHE at the 40% threshold, while all patients (100%) experienced CHE at both the 25% and 10% thresholds, indicating a substantial financial burden driven largely by medical expenses.

### 3.4 Sensitivity Analysis of Cost Drivers

The sensitivity analysis showed that 100% of patients experienced CHE when direct medical and indirect costs were increased by 5% relative to total costs, indicating that even small increases in healthcare‐related costs substantially exacerbate the financial burden on patients.

### 3.5 Determinants of CHE

Table 4 presents the results from logistic regression models examining the predictors of incurring CHE at three thresholds (10%, 25%, and 40%) for both total and direct healthcare costs. At the 25% threshold for total cost, individuals aged 30–39, 40–49, and 50–59 were significantly less likely to experience CHE compared to the reference group, with marginal effects of −0.480 (95% CI: −0.826, −0.135), −0.348 (95% CI: −0.639, −0.057), and −0.547 (95% CI: −0.854, −0.241), respectively. A similar pattern was observed for direct cost at the 40% threshold, where all age groups from 30 to 59 and those aged 60 and above had significantly lower probabilities of experiencing CHE.

**Table 4:** Marginal effects from logistic regression predicting catastrophic health expenditure (CHE) at 10%, 25%, and 40% thresholds for total and direct costs.

	Total cost			Direct cost		
Variables	CHE ≥ 10%	CHE ≥ 25%	CHE ≥ 40%	CHE ≥ 10%	CHE ≥ 25%	CHE ≥ 40%
Age						
30–39	Not estimable	−0.480** (–0.826, –0.135)	−0.433 (–1.056, 0.190)	Not estimable	–0.454 (–0.950, 0.043)*	–0.746*** (–0.965, –0.526)
40–49	Not estimable	−0.348** (–0.639, –0.057)	−0.284 (–0.890, 0.323)	Not estimable	–0.312 (–0.761, 0.138)	–0.706*** (–0.857, –0.555)
50–59	Not estimable	−0.547*** (–0.854, –0.241)	−0.379 (–1.000, 0.241)	Not estimable	–0.519 (–0.975, –0.063)**	–0.758*** (–0.893, –0.623)
Age 60+	Not estimable	−0.104 (–0.445, 0.237)	−0.096 (–0.756, 0.564)	Not estimable	–0.215 (–0.705, 0.274)	–0.652*** (–0.841, –0.464)
Gender						
Male	0.024 (–0.137, 0.185)	−0.120 (–0.277, 0.037)	−0.008 (–0.161, 0.144)	–0.005 (–0.209, 0.199)	–0.095 (–0.260, 0.070)	0.023 (–0.120, 0.165)
Education						
Primary education	Not estimable	−0.309** (–0.591, –0.026)	−0.202 (–0.511, 0.107)	Not estimable	–0.136 (–0.471, 0.199)	0.009 (–0.258, 0.277)
Secondary education	Not estimable	−0.358** (–0.622, –0.094)	−0.139 (–0.456, 0.178)	Not estimable	–0.185 (–0.521, 0.151)	–0.092 (–0.367, 0.182)
Tertiary education	Not estimable	−0.255 (–0.557, 0.047)	−0.144 (–0.508, 0.220)	Not estimable	–0.199 (–0.575, 0.178)	–0.086 (–0.407, 0.235)
Employment						
Self‐employed	0.077 (–0.100, 0.255)	0.297*** (0.084, 0.509)	0.259** (0.057, 0.461)	0.047 (–0.183, 0.276)	0.271 (0.043, 0.500)**	0.139 (–0.040, 0.318)
Unemployed	−0.148 (–0.736, 0.439)	0.357** (0.021, 0.694)	0.634*** (0.346, 0.921)	–0.107 (–0.642, 0.428)	0.461 (0.121, 0.800)***	0.422*** (0.121, 0.722)
Marital status						
Married	0.107 (–0.218, 0.432)	0.142 (–0.111, 0.395)	−0.112 (–0.364, 0.140)	0.338 (–0.114, 0.789)	0.074 (–0.194, 0.343)	–0.148 (–0.401, 0.105)
Single	0.051 (–0.387, 0.489)	−0.139 (–0.523, 0.245)	−0.294 (–0.651, 0.063)	0.133 (–0.499, 0.765)	–0.217 (–0.596, 0.162)	–0.400*** (–0.639, –0.160)
Widowed	Not estimable	−0.086 (–0.395, 0.224)	−0.314** (–0.581, –0.048)	Not estimable	–0.004 (–0.326, 0.318)	–0.240* (–0.513, 0.034)
Insurance status						
NHIS (Yes)	Not estimable	−0.259** (–0.468, –0.050)	−0.254** (–0.501, –0.008)	Not estimable	–0.299** (–0.546, –0.053)	–0.326** (–0.601, –0.051)
Observations	61	118	118	61	118	118
Prob > chi^2^	0.817	0.0001	0.0001	0.418	0.0004	0.0030

*Significant at 10%.

** Significant at 5%.

*** Significant at 1%.

Education level also showed a negative association with CHE. At the 25% threshold (total cost), both primary and secondary education levels were associated with reduced likelihood of CHE, with marginal effects of −0.309 (95% CI: −0.591, −0.026) and −0.358 (95% CI: −0.622, −0.094), respectively.

Employment status was positively associated with CHE. Self‐employed and unemployed individuals had higher probabilities of CHE at both the 25% and 40% thresholds. NHIS enrollment was associated with a significantly lower likelihood of CHE at 25% and 40% thresholds for both total and direct cost models.

## 4 Discussion

This study assessed the economic burden of diabetes management among patients receiving care at the Ho Teaching Hospital in the Volta Region of Ghana. The results highlighted a considerable economic burden of diabetes on patients, with an average monthly treatment cost of USD21.25, translating to approximately USD255.05 per year. Although this estimate is slightly lower than the USD 290.44 reported in northern Ghana [11], it exceeds the mean annual cost of USD 194.09 (GHS 540.35) identified in a recent Ghanaian scoping review [12]. These variations likely reflect differences in study periods, facility‐level practices, cost components included, and local price dynamics, underscoring the contextual nature of diabetes care costs even within the same health system.

Direct medical costs (USD18.71 per visit) constituted the dominant share of total cost, accounting for 88% of the overall total cost, predominantly driven by the cost of medications (67.17%). These results align with studies in Ghana and other sub‐Saharan African settings, which have consistently identified medicines as the primary driver of diabetes‐related expenses [11, 12, 22]. Despite high enrollment in the NHIS, patients still incurred substantial OOP expenses. This indicates that NHIS coverage may be insufficient to fully protect against the financial risks associated with chronic disease care, a challenge also highlighted in other recent Ghanaian studies [11, 12, 22]. In the Ghana Care Diabetes project, the direct cost of managing T2DM and hypertension comorbidity constituted 94% of the monthly economic cost of care, and the median direct cost of care was US$19.30 (IQR:10.55–118.88) [23]. The high cost of OOP payments has implications for adherence to medication and adds to the financial and psychosocial distress of households in which a member has diabetes.

Although indirect costs related to productivity loss were relatively low in monetary terms, the associated time burden was substantial. Patients spent considerable time traveling to health facilities and waiting for services, representing hidden costs that disproportionately affect economically active individuals. Evidence from diverse settings indicates that prolonged travel and waiting times discourage routine follow‐up and continuity of care, thereby increasing the risk of poor disease control and complications [24, 25, 26, 27]. These findings highlight the need for health system innovations, including decentralized care and telemedicine, to reduce time‐related barriers and improve access to chronic disease services.

A striking finding of this study is the high prevalence of CHE among patients. Using total treatment costs, all patients experienced CHE at the 10% threshold, nearly all (95.79%) at the 25% threshold, and over four‐fifths at the 40% threshold. Sensitivity analyses further demonstrated that even modest increases in direct medical and indirect costs led to universal CHE, underscoring the fragility of household financial resilience. These findings are consistent with previous studies in Ghana that have reported a high incidence of CHE despite NHIS enrollment [28, 29]. These findings reinforce growing evidence that chronic disease care in Ghana remains largely financed through household income, exposing patients to severe financial risk despite health insurance coverage. The continued reliance on household income to finance healthcare poses significant risks, including delayed care‐seeking, treatment nonadherence, and adverse health outcomes, particularly among the most economically vulnerable.

Multivariable analysis identified age, employment status, and insurance coverage as key determinants of CHE. Individuals aged 40–59 years were less likely to incur CHE compared with younger adults, possibly reflecting greater income stability or household coping mechanisms in this age group. In contrast, self‐employed and unemployed individuals faced higher probabilities of CHE, highlighting the vulnerability of those in informal or unstable employment. NHIS enrollment was associated with a reduced likelihood of CHE, confirming its partial protective effect, although not sufficient to prevent catastrophic spending entirely. These findings are consistent with regional evidence identifying unemployment, lack of insurance, and socioeconomic vulnerability as major predictors of CHE in sub‐Saharan Africa [30, 31].

Multivariable analysis identified age, employment status, and insurance coverage as key determinants of CHE. Individuals aged 40–59 years were less likely to incur CHE compared with younger adults, possibly reflecting greater income stability or household coping mechanisms in this age group. In contrast, self‐employed and unemployed individuals faced higher probabilities of CHE, highlighting the vulnerability of those in informal or unstable employment. NHIS enrollment was associated with a reduced likelihood of CHE, confirming its partial protective effect, although not sufficient to prevent catastrophic spending entirely. These findings are consistent with regional evidence identifying unemployment, lack of insurance, and socioeconomic vulnerability as major predictors of CHE in sub‐Saharan Africa [28, 30, 31].

## 5 Limitations of the Study

First, our study relied on self‐reported previous month data for direct costs (like medication, lab tests, and consultation fees). While this approach is susceptible to recall bias, we believe its impact was minimal since these are recurring expenses that patients are likely to remember.

Second, we estimated productivity losses using Ghana's national minimum wage. This might not fully capture the actual income lost, especially since some respondents are self‐employed. Additionally, this study estimated indirect costs based solely on time lost during hospital visits, excluding productivity losses from diabetes‐related absenteeism on nonclinic days. While this may have led to an underestimation of total indirect costs, the approach remains reasonable in this context. Most respondents were self‐employed, with flexible work schedules and limited formal sick leave, making it difficult to accurately quantify time lost outside clinic days. Consequently, time spent attending hospital visits represents the most consistent and reliably reported component of productivity loss [23, 32], and its use is unlikely to substantially bias the overall findings.

Third, the study was conducted in a tertiary hospital setting, where specialized diabetes care is associated with higher healthcare costs than services at district or primary levels. Consequently, the cost estimates and levels of CHE may not be generalizable to lower levels of care. The focus on Ho Teaching Hospital was deliberate, as it serves as a referral center for patients with complex or long‐standing diabetes requiring specialized services not routinely available elsewhere, thereby capturing the upper‐bound economic burden of advanced diabetes care.

Finally, household income was proxied using the national minimum wage because of the predominance of informal employment and the lack of reliable income data. Although this approach is commonly applied in similar settings, it may have resulted in some misclassification of true income levels. Consequently, estimates of CHE may have been overestimated for individuals whose actual incomes are substantially higher than the minimum wage and underestimated for those earning below this threshold. Nevertheless, given that most respondents are employed in the informal sector, the use of this proxy is unlikely to have substantially affected the study findings or the reflection of real‐world conditions.

Despite these limitations, a key strength of this study is its comprehensive approach to estimating both direct and indirect costs. This provides a more holistic understanding of the economic burden of diabetes, going beyond just medication and hospital visit expenses.

## 6 Conclusion

This study adds to the growing evidence that diabetes care in Ghana places a severe and often catastrophic economic burden on patients. Despite high enrollment in the NHIS, patients receiving care at the Ho Teaching Hospital face substantial financial strain, with most experiencing CHE. Medications were the main cost driver, while time losses contributed considerably to the hidden costs of care. Strengthening NHIS benefits for diabetes services at tertiary hospitals and adopting service delivery models that reduce time and transport costs are essential to mitigating the financial burden of T2DM care in Ghana.

## Author Contributions

**Maxwell Ayindenaba Dalaba:** conceptualization, investigation, writing – original draft, methodology, validation, visualization, writing – review and editing, formal analysis, project administration, data curation, supervision, resources. **Phidelia Theresa Doegah:** conceptualization, methodology, validation, visualization, writing – review and editing, formal analysis, data curation, supervision. **Mustapha Immurana:** conceptualization, methodology, validation, visualization, writing – review and editing, formal analysis, data curation. **Robert Kaba Alhassan:** writing – review and editing, formal analysis, validation, visualization. **Paul Welaga:** validation, visualization, writing – review and editing, formal analysis. **Michel Adurayi Amenah:** validation, visualization, writing – review and editing, formal analysis. **Isaiah Awintuen Agorinya:** validation, visualization, writing – review and editing, formal analysis. **Martin Amogre Ayanore:** validation, visualization, writing – review and editing, formal analysis. **Paul Amuna:** validation, visualization, writing – review and editing. **William Kofi Bosu:** validation, visualization, writing – review and editing.

## Funding

The authors have nothing to report.

## Ethics Statement

Ethical approval was obtained from the University of Health and Allied Sciences Research Ethics Committee [UHAS‐REC A.11 [22] 23–24]. Permission from the Ho Teaching Hospital was obtained before data collection. Written informed consent was obtained from respondents before interviews.

## Conflicts of Interest

The authors declare no conflicts of interest.

## Transparency Statement

The lead author Maxwell Ayindenaba Dalaba affirms that this manuscript is an honest, accurate, and transparent account of the study being reported; that no important aspects of the study have been omitted; and that any discrepancies from the study as planned (and, if relevant, registered) have been explained.

## Data Availability

The data that support the findings of this study are available from the corresponding author upon reasonable request.
